# Mobile phones and their impact on the socioeconomic development of rural women in Peru

**DOI:** 10.12688/f1000research.152716.1

**Published:** 2025-02-21

**Authors:** Lindon Vela Meléndez, Sergio Tobón, Yefferson Llonto Caicedo, Enaidy Reynosa Navarro

**Affiliations:** 1 Escuela de Economía, Universidad Nacional Pedro Ruiz Gallo, Lambayeque, Lambayeque, 14013, Peru; 2 Dirección, Centro Universitario CIFE, Cuernavaca, México, 62140, Mexico; 3 Facultad de Ciencias de la Salud, Universidad Cesar Vallejo, Trujillo, La Libertad, 13001, Peru

**Keywords:** Mincer equation, cell phones, women, rural area, income, digital gap

## Abstract

**Background:**

The study examines the impact of mobile phones on the socioeconomic development of rural women in Peru, using data from the National Household Survey from 2016 to 2022. Previous studies have highlighted the role of information and communication technologies (ICT) in empowering rural women and reducing the digital gender gap.

**Method:**

A quantitative approach and a correlational design were applied. The Mincer equation adjusted with the Heckman method was used to estimate the effect of mobile phone use on the income of rural women, considering socioeconomic and demographic variables. The sample included between 1,436 and 1,568 rural Peruvian women per year.

**Results:**

The results indicate a positive correlation between owning a personal mobile phone and the income of rural women in the period 2016-2019. In 2018, owning a mobile phone was associated with a 39.3% increase in income levels. In 2022, this effect was 49.9%.

**Conclusions:**

It is concluded that the income of rural women in Peru is positively linked to the possession of a mobile phone, but also depends on factors such as years of education, work experience, marital status, household headship, and labor market participation. The findings support the implementation of public policies to reduce the digital gender gap and promote sustainable development.

## Introduction

Globally, the gender digital gap persists and remains high, particularly in Africa, the Arab States, Asia-Pacific, and increasingly in countries with developed urban areas. 52% of women worldwide do not have access to the internet, while the percentage for men is 42%. In Latin America, by 2019, 67% of the population had subscribed to a mobile phone service, with projections indicating that by 2025, 73% of the population, representing 484 million people, will have a mobile phone subscription, albeit with a clear gender inequality ( Union, 2019).

Rotondi et al. (2019) notes that in 17 out of 23 Latin American and Caribbean countries, rural women have shown a lower probability of owning a mobile phone. However, this gap has been gradually narrowing, decreasing from 45% of rural women owning a mobile phone in 2006 to 80% in 2017. In specific cases, such as Argentina and Brazil, mobile phone ownership has become equal between genders. The gap remains pronounced in Peru and Guatemala, while in Chile and Uruguay, the ratio of women to men owning a mobile phone is greater than one.

As observed, rural women still face persistent structural constraints across all latitudes. To foster rural development, the empowerment of rural women appears crucial, which particularly entails identifying opportunities for them in each specific context. Information and Communication Technologies (ICTs), as an effective tool, can provide opportunities to promote their empowerment. Currently, the notion persists that rural women lack the capacity to independently generate income and are generally relegated primarily to household chores and poorly remunerated work. This situation unjustly limits their access to reasonable incomes, and even more so when their voices are silenced (Terry & Gomez, 2011, como se citó en Forouzani & Mohammadzadeh, 2018).

It is important to pay attention to persistent gender inequalities in the digital world, which reflect the gender gaps present in society. This issue is relevant in the context of the agreements established in relation to the Sustainable Development Goals (SDGs), particularly SDG 5, which affirms that gender equality is not only a basic human right but also a crucial component for building a peaceful, prosperous, and sustainable world. The present study adopts the Sustainable Social Development approach as a broader construct that addresses the problem from the perspective of the complexity of the phenomenon and beyond the prevailing model’s vision ( Cárdenas & Herrera-Meza, 2019; Santoyo-Ledesma, 2019). This study focuses on the concepts of wage equality in the labor market, assuming a stance that considers women as fundamental actors in the family and society, requiring equitable conditions to fulfill their roles.

The sustainable social development approach requires generating the necessary inputs to address phenomena using complex thinking and socio-formation, which necessitates building a transdisciplinary process centered on collaborative action ( Luna-Nemecio, 2019) or the construction of public policies that emphasize the participation of actors for the development of talent in individuals, communities, and organizations, seeking to overcome the problem with inclusion and the use of digital technology ( Luna-Nemecio, 2019; Luna-Nemecio et al., 2020; Tobon & Luna-Nemecio, 2021; Tobón & Vélez-Ramos, 2020).

In this context, the importance of the Santiago Commitment is highlighted, adopted by the member states of the Economic Commission for Latin America and the Caribbean (ECLAC) at the XIV Regional Conference on Women in Latin America and the Caribbean in 2020. This agreement holds significant value as a tool in the region to address the underlying causes of gender inequalities and provide a short, medium, and long-term response to the pandemic ( ONU-Mujeres, 2021).

Regarding the mechanisms to implement the commitments, in order to promote sustainable development in gender issues, it is necessary to recognize that ICTs have the potential to improve the interaction between governments and rural populations, fostering knowledge-based local economic development ( Forouzani & Mohammadzadeh, 2018).

One of the elements of ICTs that has widespread dissemination and use worldwide are mobile phones. In this regard, it is considered that: The diffusion of ICTs has had a significant impact on the lives of the poor, especially since the emergence and widespread use of mobile phones. In Latin America, mobile subscription penetration rates range from 37% in Mexico to 77% in Costa Rica, being the lowest and highest rates, respectively ( Alderete, 2019, p. 2).

Regarding mobile phones ( Stein & Mesfin, 2021), consider that the gender digital gap is also a matter of ownership and use of mobile phones for business and to obtain positions that can empower women in business. Further research should investigate whether the provision of mobile phones and training of women entrepreneurs in the use of mobile phones for business can lead to female empowerment and, therefore, eliminate or reduce the observed digital gender discrimination.

There are various studies that confirm the importance of mobile telephony as a mechanism for internet access and, consequently, communication among actors in the value chain of rural women’s agricultural organizations, access to health services, financial inclusion, and, in general, the empowerment of rural women, as it also promotes political participation and affirms culture and identity ( Alderete, 2019; Angioloni et al., 2018; Haenssgen, 2019).

In Peru, during the period 1960-2019, the rural population has shown a decreasing trend, representing less than one-fifth of the population, along with Bolivia, Peru, Mexico, Brazil, and Colombia. However, it has also demonstrated a change in the rural poverty rate, which has decreased from 68.4% in 2001 to 41.4% in 2017. In terms of inequalities in the development of digital skills, it has led to addressing the rural digital gap from the factors of supply, demand, and institutional aspects, requiring the formulation of policies for the development of telecommunications in rural areas of Peru ( Aguero y Barreto, 2012; Barrantes et al., 2020; Fort et al., 2016; Ingutia y Sumelius, 2022).

In the case of Peru, there has been a growing deployment of telecommunications infrastructure in rural areas in recent years. In 2017, households with access to mobile telephony in rural areas represented 77.8%; while in 2018, it increased by 2.9%. In 2019, the rural households that increased access to mobile phones was 0.9%, and in 2020, 82.4% of rural households had access to mobile phones, increasing by 0.5%, presenting a gap of 17.6%.

In this context, the expansion of mobile phones has been important, becoming an almost essential tool in the world of information and communication technologies; but women’s access to rural connectivity has a strong impact on the socio-economic reality of the countryside, hence the objective of the research is to quantify the relationship between the use of mobile phones and the income of rural women in Peru in the period 2016-2022; for which the National Household Survey is used using the MINCER equation, considering socioeconomic variables, in order to answer the research question: Is there a relationship between the use of mobile phones and the income of rural women in Peru in the period 2016-2022?

## Literature review

At the international level, it is observed that there are still high gender gaps with respect to access to mobile phones. There are various barriers, from cultural, gender, attitude, economic and social barriers that do not allow balancing around access to information and communication technologies and the benefits in equal opportunities generated by access to mobile phones. Digital information and communication technologies such as mobile phones are contributing to development in the rural sector, mainly through women’s empowerment, since the possession of mobile phones allows women not only to streamline their productive processes in the field and their greater connection with the market, but also allows them to access socioeconomic services, access medical care, financial services, family reporting services and planning.

Hence, Scott et al. (2021) in their study of India consider that the digital gender gap has become a greater disadvantage for women in rural areas. Women in this part of the world still face patriarchal thinking that leads to becoming a significant cultural barrier. These barriers generate losses of employment opportunities, access to health, access to public services, citizen participation and access to financial services, becoming a clear disadvantage for women.

Kenny and Regan (2021) consider that although the use of mobile phones has been significantly deployed in the agricultural sector in Ireland, the lack of internet access is also evident. In the case of people who have access to technologies, it has not only generated flexibility, empowerment, stress reduction, time efficiency and communication development, but also data generation networks allow for greater competitiveness in the sector. Likewise, in rural communities such as Zimbabwe in Africa, there is also a connectivity gap in telecommunications due to the deficient digital infrastructure and the complex socio-political environments observed as an indicator of coverage in access to mobile telephony.

In this way, Das et al. (2020) points out that women in developing countries face the problem of access to mobile phones, computers and the internet. The lack of access to mobile telephony affects the socio-cultural aspects of rural areas, which leads to an inequality index in the use and access of information and communication technologies. This scenario generates inequality in various countries that leads to an imbalance of empowerment between rural and urban areas.

Rahim et al. (2020) in their research addresses the access to a mobile phone by rural women in two districts of Pakistan, where they find that 80% of women have a mobile phone and 63% of them have skills for its use, evidencing the important strategy of using mobile phones in the daily activities of rural women, generating socioeconomic development by providing skills in rural businesses.

For Li et al. (2022), the use of smartphones has led to changing farmers’ behavior, mainly due to access to information, smart agricultural development, environmental mitigation, and farmer satisfaction with crop productivity results. Hence, Holden et al. (2021) consider that mobile phones have positioned women in terms of rural business group leadership, resulting in 119 young business groups, 32% of which were women; however, male members were more likely to be leaders than female members. Considering that the digital gap was considered an issue of ownership in the use of mobile phones to exercise rural businesses and develop a rural businesswoman.

Mobile phones have become a vitally important tool for rural development in East African societies, where from the theoretical perspective of social networks, social ties have been developed based on greater use of mobile phones that have contributed to the productive process of their agricultural crops, but which face challenges in terms of security given the scams that would also be generated via mobile telephony, leading to a security policy in terms of mobile telephony ( Baird et al., 2021).

The increasing use of mobile phones and internet technology has considered a considerable technological revolution in modern agriculture, given that the study in four districts of the Khyber Pakhtunkhwa (KPK) province of Pakistan showed that after a regression model, a significant impact was evidenced in the development of commercial channels, productivity, family income and effectiveness in their marketing channels that has a positive impact on improving the productive capacity of farmers ( Khan et al., 2022; Prete & Calleja, n.d.).

For Ujakpa et al. (2021) reaffirms the benefits that mobile telephony has brought about, generating optimal decisions in the face of the problem of information asymmetries in agricultural markets; considering 140 rural participants in the applied questionnaire, it was obtained that the use of the mobile phone generates value and economic benefits in practice given the greater information generated by transactions in the input markets, access to meteorological information and banking services, providing agricultural information for efficiency in rural areas.

Finally, Mwalupaso et al. (2019) considers that mobile technology has played a fundamental role in generating useful information for agricultural producers, with the development of skills in women being vital, due to the development of skills through flexibility in their use, infrastructure requirements and the affordability of telephony, mainly covering their access in rural areas; this has led not only to the computerization of the rural aspect, but also to a change in the behavior of small producers. For this, producers need to adopt skills for the formation of business networks, leading to a reconfiguration of their agricultural crops, the use of agricultural inputs, the harvesting, transportation and marketing process, with the mobile phone being a decision support system ( Shaukat et al., 2014).

## Methods

From the literature review, it is observed that the importance of the diffusion of cellular telephony in the empowerment of rural women has been addressed methodologically in the great majority of studies by the quantitative method and fundamentally by regressions considering socioeconomic and demographic variables ( Abdul-Rahaman & Abdulai, 2020; Ajide et al., 2022; Alderete, 2019; Ali et al., 2019, 2020; Angioloni et al., 2018; Forouzani & Mohammadzadeh, 2018; Stein & Mesfin, 2021).

### Methodological design

The methodological approach considers the traditional quantitative approach and correlational type of research, given that it seeks to analyze the effect of the use of mobile phones on the income of women in rural areas in Peru, for which the National Household Survey for the years 2016-2022 is used.

The ( Mincer, 1996) model is used, where from this model the change in income resulting from the socioeconomic explanatory variables such as mobile phone ownership, years of education, work experience and the individual’s own characteristics is quantified.

### Procedure

The data were obtained from the National Household Survey data files for the years 2016 to 2022 from module 02 (Characteristics of Household Members)-Enaho01-2022-200 and module 05 (Employment)-Enaho01-2022-500 published on the website of the National Institute of Statistics and Informatics.

The estimation of the change in income for rural women in Peru from the use of mobile phones uses the ( Mincer, 1996) model considering the explanatory variables of years of education (S), work experience (Exp), and socioeconomic characteristics of the individual (L) to determine the standardized level of income (W).

Leaving the equation 1 of the Mincer model expressed as detailed below:

$$W=\beta_{0}+\beta_{1}S+\beta_{2}\mathrm{Exp}+\beta_{3}\mathrm{Exp}2+\beta_{4}L+\mu_{1}$$ (1)

Although equation 1 is used to quantify the effect of education and work experience on income, however, this equation with the aforementioned explanatory variables would be incomplete.

Hence, ( Mincer, 1996) considers the explanatory factors in two groups: both labor aspects and aspects specific to individuals, the latter being relevant in the research on the use of mobile phones by rural women in Peru.

To correct for selection bias in the data, the approach proposed by Heckman (1979) was employed through the use of two simultaneous equations incorporating dependent variables related to reported income and unreported (reservation) income. This method begins with the estimation of a probit model of labor market participation. The equations used are described below:

$$\text{Part}=\alpha_{0}+\alpha_{1}*S_{i}+\alpha_{2}*{\mathrm{Exp}}_{i}+\alpha_{3}*\mathrm{Exp}2_{i}+\alpha_{4}*{\text{Jefa}}_{i}$$ (2)

In the second equation, “Part” is a binary variable that is set to one if person i is active in the labor market, or zero if not. “S” represents the years of formal education, “Exp” are the years of potential experience (as a proxy for work experience), “Exp2” is experience squared, and “Jefa” is a binary variable that is set to one if person i is the head of the household, or zero if not.

$$\mathrm{LnW}=\theta_{0}+\theta_{1}*S_{i}+\theta_{2}*{\mathrm{Exp}}_{i}+\theta_{3}*\mathrm{Exp}2_{i}+\theta_{4}*{\mathrm{EC}}_{i}+\theta_{5}*{\text{UCPR}}_{i}+\theta_{8}*\lambda_{i}$$ (3)

The third equation is based on the explanatory variables used in the Mincer model, additionally adding control variables. The EC variable is a binary indicator that reflects an individual’s marital status, taking the value of one if married and zero otherwise. Furthermore, the lambda variable (λ), also known as the inverse Mills ratio (1926), is added to complement the approach proposed by Heckman (1979). The research has incorporated an additional explanatory variable related to the use of mobile phones, categorizing the possession of this technological device. UCPR is a binary variable that takes the value of one if the individual i has their own mobile phone and zero if they do not.

The model for estimating the influence of mobile phone ownership on the income of rural women includes a known problem called selection bias, which is generated because the sample for estimating the wage equation is not random, given that an attempt is made to estimate a wage equation with employed rural women and information is excluded on the variables influencing women who do not report wages. At the same time, those factors affecting wages also have an impact on the decision to participate in the labor market. This correlation between unobserved effects in the determination of wages and labor force participation generates the endogeneity problem. This problem can result in biased and inconsistent inferences, even if the sample used is of an adequate size.

The two-step method proposed by Heckman (1979) was used to correct for selection bias in the data. This involves incorporating the inverse Mills ratio variable in the wage equation, after having estimated the probability of labor market participation. The presence of a statistically significant parameter associated with the inverse Mills ratio indicates the existence of sample selection bias and that the problem has been effectively corrected.

The variables used for estimating the model are detailed in Table 1.

**Table 1.** Variables used in the model.

Variables	Type	Description	Unit
W	Dependent	Monthly income	Peruvian Soles
UCPR	Explanatory note of interest	Own mobile phone	1/0 Dummy
Part	Explanatory Control	Labour market participation	1/0 Dummy
S	Explanatory Control	Years of Education	Years
Exp	Explanatory Control	Work experience	Years
Exp2	Explanatory Control	Work experience squared	Years squared
EC	Explanatory Control	Marital status	1/0 Dummy
Jefa	Explanatory Control	Head of household	1/0 Dummy

Note: In the following, only acronyms will be used to specify equations.

### Sample

The sample comprises a total of 1,452 Peruvian rural women in 2016; on the other hand, 1,436 Peruvian rural women in 2017; in 2018 the sample amounted to 1,568 rural women; in 2019 the sample amounted to a total of 1,523 rural women as well as in 2020 corresponding to the National Household Survey, considering inclusion and exclusion criteria.

Inclusion criteria: Women aged 14 and over who are in the agricultural sector of the National Household Survey from 2016 to 2020.

Exclusion criteria: People aged 14 and over who are in a sector other than agriculture in the National Household Survey from 2016 to 2020.

### Data analysis

The Mincer (1996) model adjusted with the ( Heckman, 1979) method was used to estimate the effect of mobile phone use on the income of women in rural areas in Peru, using Eviews 9 A license for academic institutions is held under the name of Lindon Vela Meléndez: Lite Licensing Information: Serial Number: Q1208886 - D49010AF - 9D854485 Download Link: http://www.eviews.com/download/student12 software for data analysis.

## Results

In 2020, from the sample of rural women in Peru, the average salary amounted to 452.44 soles (as observed in Table 2), which is below the minimum vital salary of 930 soles; however, it has shown an increasing trend compared to 2016 when the average salary was 259.57 soles; in 2017 it reached an average salary of 277.72 soles; in 2018 it reached a salary of 344.34 soles and in 2019 the average salary fell to 332.59 soles.

**Table 2.** Descriptive statistics of non-dichotomous explanatory variables.

Variables	Obs.	Average	Standard deviation	Min.	Máx.
**W**	1523	452.44	508.19	3	7853.17
**S**	1523	4.74	4.54	0	17
**Exp**	1523	9.94	11.16	1	59

Note. Abbreviations used: “Obs.” = Number of observations, “Std. Dev.” = Standard Deviation, “Min.” = Minimum, “Max.” = Maximum. Due to the type of information collected, the geometric mean is used.

With respect to years of education, there is an average of 5 years, indicating that the average rural woman in Peru completed primary education; however, this average has decreased compared to 2016 when it was 6 years; in 2017 the years of study increased to 9 years as in 2018 and 2019.

Regarding work experience, the average is 10 years, the same as in 2018 and 2019; while in 2017 the average was 9 years and in 2016 the average was 6 years.

To properly model a social or economic reality, it is essential to incorporate dichotomous variables such as participation, ownership, and marital status. These variables enable the detection of significant differences that would not be observable with continuous variables ( Acharya et al., 2022).

In 2020 (as observed in Table 3), 67% of the sample participates in the labor market, 19% are not single, 69.5% use their own mobile phone, 24% use a family mobile phone, and 60.9% are not heads of household, so the individual under study who participates in the labor market is not single, uses their own mobile phone and is not the head of household.

**Table 3.** Descriptive statistics of dichotomous explanatory variables.

Variable	1	0
Part	67%	33.%
EC	19%	81%
UCPR	69.5%	30.5%
Jefa	39.1%	60.9%

Note. Meanings of numbers used: “1” = Affirmative answer, “0” = Negative answer.

In Table 4 (1), the estimation of the Mincer model, it is observed that the use of one’s own cell phone is significantly related to the income of women in rural areas of Peru in the years 2017, 2018 and 2019.

In 2016, it is observed that the labor market participation of rural women significantly depends on the years of education, with an additional year of education level being associated with a 3.2% change in the probability of participation, as well as the household head status being associated with a 17% change in the probability of participating in the labor market. In the Mincer equation, it is observed that, in this year, the years of education and the possession of a mobile phone by rural women in Peru are associated with changes in the income level of rural women. An additional year of education is associated with a 7.8% increase in income and the possession of one’s own mobile phone is associated with a 13.7% change in income, however, it is reported that the latter association is not significant, since the p-value is 0.1537.

In 2017, the labor market participation of rural women significantly depends on the years of education, with an additional year of education level being associated with a 3% change in the probability of participation, the other variables are not significant. In the Mincer equation, it is observed that, in this year, years of education, work experience and possession of one’s own mobile phone significantly influence wage levels. In the case of possession of one’s own mobile phone (i.e. the change from not having access = 0, to having access to a mobile phone = 1), it is associated with a 16.4% change in income level, considering that:

$\theta_{5}=\frac{\mathrm{\partial lnW}}{\partial \;\text{UCPR}}=$ The movement of UCPR from 0 to 1 is associated with a change of 100* θ5% in LnW.

In 2018, it is observed that the labor market participation of rural women significantly depends on the years of education and the household head status. In the Mincer equation, it is observed that, in this year, years of education, work experience, marital status and possession of one’s own mobile phone significantly influence wage levels. In the case of possession of one’s own mobile phone, it is associated with a 39.3% change in income level.

In 2019, the labor market participation of rural women significantly depends on the years of education and work experience. In the Mincer equation, it is observed that, in this year, years of education, work experience, marital status and possession of one’s own mobile phone significantly influence wage levels. In the case of possession of one’s own mobile phone, it is associated with an 18.2% change in income level.

In 2020, it is observed that the labor market participation of rural women only depends on the household head status. In the Mincer equation, it is observed that, in this year, years of education and work experience significantly influence wage levels. In the case of possession of one’s own mobile phone, it does not appear significant, which could be explained by labor market distortions as a consequence of the COVID-19 pandemic, however, this would be part of the pending agenda of the research line.

In 2021, the labor market participation of rural women only depended on work experience. In the Mincer equation, it is observed that, in this year, experience and marital status significantly influence wage levels. In the case of possession of one’s own mobile phone, it does not appear significant, similar to what occurred in 2020.

In 2022, the labor market participation of rural women only depended on the household head status. In the Mincer equation, it is observed that the trend is reversed and the possession of a cell phone again significantly influences wage levels. In the case of possession of one’s own mobile phone (i.e. the change from not having access = 0, to having access to a mobile phone = 1), it is associated with a 49.9% change in income level.

To date (February 2024), the National Household Survey of Peru does not have the annual figures for 2023, so it has not yet been incorporated into this study ( Table 4 (2)).

**Table 4 (1).** Bias correction and estimation of the Mincer Model 2016-2020.

Dependent variable	Years									
	2016		2017		2018		2019		2020	
	Part	LnW	Part	LnW	Part	LnW	Part	LnW	Part	LnW
	(Probit Model)	(Mincer Model)	(Probit Model)	(Mincer Model)	(Probit model)	(Mincer Model)	(Probit model)	(Mincer Model)	(Probit model)	(Mincer Model)
Explanatory variables	Coef.	Coef.	Coef.	Coef.	Coef.	Coef.	Coef.	Coef.	Coef.	Coef.
Constante	0.03674	4.502820	0.189479	5.534884	0.097831	5.287631	0.298845	5.314378	0.635906	5.313183
	0.5693	(0.0000)**	0.0676	(0.0000)**	0.3512	(0.0000)**	(0.0058)*	(0.0000)**	(0.0002)**	(0.0000)**
S	0.031801	0.078647	0.030002	0.025259	0.050536	0.033629	0.040655	0.032591	-0.011819	0.030124
	(0.0000)**	(0.0000)**	(0.0011)**	(0.0329)*	(0.0000)**	(0.0022)**	(0.0000)**	(0.0050)**	(0.2313)	(0.0012)**
Exp	-0.001203	0.000440	-0.003567	0.026064	0.010951	0.021344	-0.0355	0.057844	-0.003986	0.028391
	0.8974	0.9677	0.6977	(0.0386)*	0.1966	(0.0370)*	(0.0000)**	(0.0000)**	0.625	(0.0001)**
Exp ^2^	0.0000561	-0.000184	-0.00004	-0.000599	-0.000324	-0.000334	0.00067	-0.000896	0.0000722	-0.000531
	0.8293	0.5483	0.8709	-0.094	0.1234	0.2165	(0.0014)**	(0.0009)**	0.5268	(0.0000)**
EC	-	0.091391	-	-0.175048	-	-0.243467	-	-0.161104	-	-0.01321
		0.2806		-0.062		(0.0045)**		0.0774		0.8632
UCPR	-	0.137594	-	0.164371	-	0.393083	-	0.182038	-	-0.008554
		0.1537		(0.0523)*		(0.0000)**		(0.0269)*		-0.9023
Jefa	0.176996	-	-0.018056	-	-0.140377	-	-0.022814	-	-0.277375	-
	(0.0423)*		0.7721		(0.0261)*		0.7114		(0.0000)**	
Lambda (λ)	-	0.149006	-	0.365768	-	0.312036	-	0.33058	-	-0.055116
		(0.0009)**		(0.0000)**		(0.0000)**		(0.0000)**		(0.0314)*
Observaciones	1452	1452	1436	1436	1568	1568	1523	1523	1523	1523

**Table 4 (2).** Bias correction and estimation of the Mincer Model 2021-2022.

Dependent variable	Years			
	2021		2022	
	Part	LnW	Part	LnW
	(Probit Model)	(Mincer Model)	(Probit Model)	(Mincer Model)
Explanatory variables	Coef.	Coef.	Coef.	Coef.
Constant	0.5375	5.797240	0.579728	4.710590
	(0.0000)**	(0.0000)**	(.0.0001)**	(.0.0000)**
S	0.0125	0.000287	0.0068	0.020262
	(0.0989)	(0.9755)	(.0.4902)	(.0.0554)
Exp	-0.038944	0.056898	-0.00448	0.000580
	(0.000)**	(0.0000)**	(.0.5298)	(.0.9462)
Exp ^2^	0.000685	-0.000933	0.000122	0.0000403
	(0.0011)*	(0.0000)**	(.0.2685)	(.0.7602)
EC	-	-0.2567726	-	-0.320564
		(0.0044)*		(.0.0003)**
UCPR	-	0.027508	-	0.499796
		(.0.7063)		(.0.0000)*
Jefa	0.077246	-	-0.323349	-
	0.2208		(0.0000)**	
Lambda (λ)	-	0.319863	-	0.182387
Observaciones	1523	1523	1568	1568

Note: The significant values of the Lambda (λ) parameter indicate that the selection bias problem did indeed exist and that it has been adequately corrected with Heckman’s two-stage methodology in the years 2016 to 2022. S = Years of education, Exp = Work experience, Exp ^2^ = Work experience squared, EC = Marital status, UCPR = Ownership of personal mobile phone, and Jefa = Head of Household.

In Table 5, the effects of owning a personal mobile phone on the income of rural women in Peru are analyzed. For this, the parameters of the independent variables of the multiple regression with a logarithmic dependent variable are analyzed. These regression coefficients are interpreted as effect sizes. However, there are important considerations to take into account. First, because the dependent variable is on a logarithmic scale, the effect sizes refer to changes in the ratio, rather than changes in the absolute rate. Second, it is important to keep in mind that the parameters of the independent variables are a function of the scale on which the independent variables are measured.

**Table 5.** Size and Interpretation of the Effect of the Regression Coefficient θ _5_ (UCPR Parameter).

Year	Value of the Coefficient θ _5_ (Effect Size)	P-value	Statistical significance	Meaning of the Effect of Going From 0 to 1 on the Dummy Variable
2016	0.137594	0.1537	Not significant	Associated with a 13.7% change in income level
2017	0.164371	(0.0523) *	Significant	Associated with a 16.4% change in income level
2018	0.393083	(0.0000) **	Significant	Associated with 39.3% change in income level
2019	0.182038	(0.0269) *	Significant	Associated with 18.2% change in income level
2020	-0.008554	-0.9023	Not significant	Distortion possibly explained by covid-19 pandemic scenario
2021	0.027508	(.0.7063)	Not significant	Distortion possibly explained by covid-19 pandemic scenario
2022	0.499796	(0.0000) **	Significant	Associated with 49.9% change in income level

Note: In the regression case, the effect size was considered to be the regression coefficient of the UCPR variable (Own mobile phone usage), ceteris paribus the other variables.

The calculated effect sizes range from weak to moderate (< 0.2 weak, between 0.2 and 0.5 moderate and > 0.5 strong).

Therefore, the effect sizes are interpreted considering a log-dummy regression in the particular case of the coefficient related to the independent variable UCPR, ceteris paribus the other variables.

## Discussion

The research results are aligned with those presented by Adegboye et al. (2022) who, using a double-censored Tobit regression with data from 81 middle-income countries, found an important relationship between equitable distribution of resources and information and communication technology (ICT) and inclusive human development (inequality-adjusted human development), concluding that in light of the findings, an equitable distribution of public goods, such as technologies, could play a fundamental role in promoting inclusive human development.

In the same vein, the research results are also consistent with those of Alam et al. (2019) who, when studying the influence of socio-demographic factors on the adoption of mobile phones in rural areas of Bangladesh and their policy implications, concluded that there is a likelihood that increasing use of mobile phones will improve the exchange of valuable information among rural households for better livelihood management and better agricultural decisions.

The findings regarding the positive relationship between cell phone use and income level between 2016 and 2019 are aligned with those of ( Zhu et al., 2022), who in the research titled “ICT Adoption, Individual Income, and Psychological Health of Rural Farmers in China,” using an econometric approach with data collected from 7,065 rural households, demonstrate that both ICT adoption and higher incomes are related to higher levels of happiness and life satisfaction, but also to lower levels of stress and loneliness. Additionally, they found that there is a positive interaction effect between ICT adoption and individual income.

On the other hand, the study results are also shown to be consistent with the findings of ( García Islachin, 2018), who in his research on the impact of the use of mobile phones on the socio-economy of rural women, finds that access to mobile telephony is related to the level of economic income of rural women in Anco Huallo Apurimac, Peru in 2015. In testing his hypothesis, he used the non-parametric chi-square test of independence, which resulted in a p-value of 0.050 < 0.05.

( Girmay Giday, 2019) finds results along the same lines as the present research. In the article entitled “Information and Communications Technology and Economic Growth in Sub-Saharan Africa: A Panel Data Approach”, they found that the increase in the adoption of mobile telephony has had a significant impact on the economic growth of the region, measured by GDP per capita, after taking into account other factors. It has been found that a 10% increase in mobile phone penetration leads to a 1.2% increase in GDP per capita. Therefore, improving access to mobile phones could play an important role in reducing poverty in the region by increasing the per capita income of the population.

Finally, authors such as ( Mwalupaso et al., 2020; Rahim et al., 2020) also agree with the results of the present research related to the importance of access to mobile telephony in Pakistan and Zambia respectively. Both studies find a positive relationship with the socioeconomic conditions of rural women.

### Strength

One of the main strengths of the study is the use of a robust and nationally representative database, the Peruvian National Household Survey. This gives solidity to the obtained results, allowing generalization of the findings to the rural female population of the country. Additionally, the use of a recognized econometric model such as Mincer’s, adjusted with the Heckman method to correct for selection bias, contributes methodological rigor to the analysis performed.

### Limitations

The study focuses solely on mobile phone possession, but does not consider other related factors such as the type of device, the contracted data plan, the digital skills of women, among other aspects that could also influence the benefits obtained from their use. There is no information on the specific use that rural women give to their mobile phones (for example, whether they use it for productive, commercial, educational purposes, etc.), which limits the analysis of the mechanisms through which this technology impacts their income. The study is limited to the Peruvian national context, so its findings may not be extrapolable to other rural contexts with different socioeconomic and technological realities. The available data ends in 2022, so potential effects of more recent events such as the political and social crisis that Peru has been going through since the end of 2022, which could have impacted the use of mobile phones and the income of the rural population during 2023, are not considered.

## Conclusions

During the analysis period 2016-2022, the average salary of rural women has shown an increasing trend, reaching an average salary of 452.44 soles in 2020, which is below the minimum vital salary. On the other hand, the educational level of rural women who have completed corresponds to primary education. Regarding work experience, the average number of years is 10 years of work experience, as in the years 2018 and 2019; while in 2017 the average was 9 years and in 2016 the average was 6 years.

In 2020, a majority of rural women in Peru, 67%, participated in the labor market. The proportion of women who were not single was 19%, while 69.5% owned a mobile phone and 24% used a family mobile phone. In addition, 60.9% were not heads of household. Therefore, the individual studied in this survey is one who participates in the labor market, is not single, owns a mobile phone, and is not the head of household.

The income of rural women is positively associated during the analysis period 2016-2019 with the ownership of their own mobile phone, only in 2020 the results differ from the theoretical expectation, this is probably due to labor distortions generated by the COVID-19 pandemic.

In the 2016-2022 analysis period, the variables that are consistent with the estimation of the Mincer model and are significant are the following: S (years of education, all years), Exp (years of work experience, except for 2016 and 2017) and the UCPR (dichotomous variable for owning one’s own phone, except for 2020 when it changes sign from what was expected).

The results of the estimates show that there is a positive relationship between the ownership of mobile cell phones by rural women in Peru and their income levels, in most years of the sample, which is also greatly aligned and consistent with studies discussed in different parts of the world, thus generating a reference for implementing public policies within the framework of the 2030 agenda and the objectives aimed at sustainable social development to reduce the gender gap and in particular the gender wage gap.

## Ethics and consent

The National Household Survey, being an instrument of a public entity of the Peruvian government, is rigorous in respecting the rights of participants by obtaining their informed consent and guaranteeing the anonymity and confidentiality of the data. In addition, ethical criteria such as respect, autonomy, justice, confidentiality and transparency in the handling of survey data are applied.

The Ethics Committee of the CIFE University Center in Mexico approved the research project, considering that measures will be implemented to protect the confidentiality and well-being of the participants. The project poses no physical or psychological risks to the participants, as it involves the analysis of an existing database and has a clear and well-defined scientific justification. The study’s objectives are relevant, and the methodology is appropriate for achieving them.

The document certifying this approval is titled Certificate of Approval from the Ethics Committee for Research at the CIFE University Center in Mexico. The approval code is CIFE-PIH-2022-412, dated April 12, 2022, signed in Cuernavaca, Morelos, Mexico.

the study is longitudinal and retrospective. The Peruvian government conducts the survey annually, and our research is strengthened by including more historical data, which provides stability to our results.

## Data Availability

- Zenodo: Processed database and results of the project Mobile phones and their impact on the socio-economic development of rural women in Peru, https://doi.org/10.5281/zenodo.12216409 ( Vela Meléndez et al., 2024). The data contains: The processed data tables of modules 2 and 5 of the National Household Survey of Peru 2016-2022 and also the results of the econometric models (Mincer Model and Part Model). Data are available under the terms of the Creative Commons Attribution 4.0 International license (CC-BY 4.0). The raw data from modules 2 and 5 of the National Household Survey of Peru, which served as the basis for the study, are accessible through the following link: https://proyectos.inei.gob.pe/microdatos/. Within the site, follow this access path: Survey consultation/Survey (ENAHO updated methodology)/Living conditions and poverty - ENAHO/Select the year/Period.
