# Supplementary material for: Mobile Phones and Their Impact on the Socioeconomic Development of Rural Women in Peru
Source: F1000Res. 2026 Oct 6;14:227. Originally published 2025 Feb 21. [Version 2] doi: 10.12688/f1000research.152716.2 (PMC13653101; doi:10.12688/f1000research.152716.2)
Supplement: Peer review history

# Reviewer report: Reviewer response for version 2

**DOI:** 10.5256/f1000research.211567.r527760

**Published:** 2026-10-10

**Authors:** Silvia Lourdes Vidal Taboada, Nilthon Pisfil Benites

**Affiliations:** 1 Lambayeque, Universidad Tecnologica del Peru, Lima District, Lima Region, Peru; 2 Universidad Tecnologica del Peru, Lima District, Lima Region, Peru

This review highlights the high relevance of the article, owing to the significant improvements made to its methodological and theoretical aspects. Furthermore, the model’s limitations are explicitly stated, thereby enhancing the study’s rigour and transparency.The comments have been addressed; therefore, approval is granted.

Is the work clearly and accurately presented and does it cite the current literature?

Yes

If applicable, is the statistical analysis and its interpretation appropriate?

Partly

Are all the source data underlying the results available to ensure full reproducibility?

Yes

Is the study design appropriate and is the work technically sound?

Yes

Are the conclusions drawn adequately supported by the results?

Yes

Are sufficient details of methods and analysis provided to allow replication by others?

Partly

Reviewer Expertise:

Economía

We confirm that we have read this submission and believe that we have an appropriate level of expertise to confirm that it is of an acceptable scientific standard.

# Reviewer report: Reviewer response for version 1

**DOI:** 10.5256/f1000research.167511.r397778

**Published:** 2025-09-05

**Authors:** Silvia Lourdes Vidal Taboada, Nilthon Pisfil Benites

**Affiliations:** 1 Lambayeque, Universidad Tecnologica del Peru, Lima District, Lima Region, Peru; 2 Universidad Tecnologica del Peru, Lima District, Lima Region, Peru

Methodological Assessment

 The study appropriately employs the Mincer equation with a Heckman selection correction, which is a relevant choice for the research question. However, several methodological aspects limit the robustness and credibility of the results. There is no evidence of alternative model specifications to test validity, such as sensitivity analyses with and without the selection correction, or the use of a Tobit model to account for censored income. The model’s global significance (adjusted R² or LR test) is not reported, nor are key econometric assumptions—multicollinearity, heteroscedasticity, and normality—evaluated.

 Although the specification is a log-linear OLS, it is unclear whether standard errors were corrected for heteroscedasticity, which is common in income equations (Cameron & Trivedi, 2005). The absence of robust errors may lead to under- or overestimation of statistical significance, affecting inference reliability. The decision to run separate cross-sectional regressions by year misses the opportunity to exploit the panel structure of the ENAHO data. Applying fixed or random effects models would allow control for unobserved heterogeneity across regions or cohorts, strengthening causal interpretation.

 The key regressor—ownership of a mobile phone—is potentially endogenous. While the Heckman procedure partially addresses selection, it does not resolve endogeneity from omitted variables such as digital skills or entrepreneurial orientation. This could bias coefficients upward or downward. The use of valid external instruments through IV/2SLS could mitigate this risk. Furthermore, the study does not explore the mechanisms through which mobile phone use may influence income—whether via access to markets, financial services, or labor opportunities—limiting the interpretative depth of the findings.

 Results Interpretation

 The results are presented clearly year-by-year, highlighting temporal trends. However, coefficient instability is evident, with the UCPR estimate ranging from 16.4% in 2017 to 49.9% in 2022. Such variability suggests model instability or the influence of unobserved shocks. The non-significant effects in 2020 and 2021 are attributed to the pandemic, yet no robustness checks or control variables are included to substantiate this claim. Consolidating the analysis into panel models could help separate structural effects from year-specific shocks.

 Some effect sizes, particularly in 2022 (0.49), are relatively large and merit further discussion of possible omitted factors. The inclusion of marginal effects interpretation—especially for binary regressors—would improve the practical relevance of the results by translating statistical findings into income estimates.

 Literature and Referencing

 While the study cites recent and geographically relevant literature on ICTs and rural development, it underutilizes specialized econometric sources directly related to the Mincer equation and the Heckman model. Foundational works such as Wooldridge (2010), Cameron & Trivedi (2005), and Card (1999) are absent, limiting the methodological grounding. The reference to Mincer (1996) lacks a complete bibliographic entry and appears to rely on an uncommon or inaccurately adapted version rather than the canonical 1974 work.

 Additionally, there is an overreliance on general ICT and rural development references that are not directly tied to the Peruvian context or to labor econometric modeling. Strengthening the literature with recent Peruvian or Latin American empirical studies would enhance contextual relevance. Integrating multiple citations into thematic syntheses—rather than listing them sequentially—would also enrich the analytical depth of the literature review.

Is the work clearly and accurately presented and does it cite the current literature?

Yes

If applicable, is the statistical analysis and its interpretation appropriate?

Partly

Are all the source data underlying the results available to ensure full reproducibility?

Yes

Is the study design appropriate and is the work technically sound?

Yes

Are the conclusions drawn adequately supported by the results?

Yes

Are sufficient details of methods and analysis provided to allow replication by others?

Partly

Reviewer Expertise:

NA

We confirm that we have read this submission and believe that we have an appropriate level of expertise to confirm that it is of an acceptable scientific standard, however we have significant reservations, as outlined above.

# Response

**Published:** 2025-09-08

**Authors:** Lindon Vela Meléndez

**Affiliations:** Lambayeque, Universidad Nacional Pedro Ruiz Gallo, Lambayeque, Lambayeque, Peru

**Response to the Comments Made**

**Comment 1**

***“The study appropriately employs the Mincer equation with a Heckman selection correction, which is a relevant approach for the research question. However, several methodological aspects limit the robustness and credibility of the results. There is no evidence of alternative model specifications to test validity, such as sensitivity analyses with and without the selection correction, or the use of a Tobit model to account for censored income. The global significance of the model (adjusted R² or LR test) is not reported, nor are key econometric assumptions evaluated: multicollinearity, heteroscedasticity, and normality.”***

 In response to this comment, the inclusion of the following paragraph is proposed at the end of the **Methodological Design** section, prior to the **Procedure**:

 Paragraph to be incorporated

 With regard to the robustness and validity of the results, it is relevant to highlight that the main focus of this study lies in analyzing the effect size of the coefficient associated with the UCPR variable, as developed in Table 5. Since the Heckman selection correction model is structured in two stages, the interpretation of global model fit through a classical coefficient of determination (R²) is of limited relevance. In the first stage (probit), the use of a traditional R² is not appropriate, as the model is estimated via maximum likelihood, and alternative measures such as likelihood statistics or pseudo-R²—whose interpretation differs substantially from the conventional R²—are more suitable. In the second stage (wage equation with correction by λ), although it is possible to compute an R², its practical utility is constrained, given that the goal is not to assess the overall goodness of fit for labor income—which is conditioned by multiple structural and contextual factors—but to accurately estimate the marginal effect of the variable of interest in wage determination. Within this framework, emphasis on the effect size of UCPR constitutes a more direct and substantive measure of the economic significance of the finding, reinforcing the empirical interpretation of the results beyond global fit metrics that do not offer additional insight into the research question.

**Comment 2**

***“Although the specification is a log-linear OLS, it is unclear whether standard errors were corrected for heteroskedasticity, which is common in income equations (Cameron and Trivedi, 2005). The absence of robust errors may lead to under- or overestimation of statistical significance, thus affecting the reliability of inference. The decision to run separate cross-sectional regressions by year misses the opportunity to exploit the panel structure of ENAHO data. Applying fixed or random effects models would allow for controlling unobserved heterogeneity across regions or cohorts, thereby strengthening the causal interpretation.”***

 The empirical design of this study is based on the specification of the Mincer equation with Heckman selection correction, applied to annual cross-sectional slices of the ENAHO dataset. This methodological choice is justified insofar as the main objective lies in estimating the magnitude of the effect of owning a personal mobile phone (UCPR) on labor income, without intending to exploit a longitudinal tracking of the same individuals over time. It is important to clarify that ENAHO, in practice, constitutes a set of **repeated cross-sections**, which does not allow for the construction of a strict individual-level panel necessary for the application of fixed or random effects models. In this sense, the Mincer-Heckman strategy is coherent and appropriate for the research question posed.

 Regarding the critique on econometric assumptions, we acknowledge that heteroskedasticity is frequently present in wage equations (Cameron & Trivedi, 2005). Thus, we accept the relevance of reinforcing the estimation with robust standard errors (White or Huber-White type) to ensure that statistical inference does not rely on restrictive assumptions regarding error variance. Although the results reported are already consistent under the Heckman estimator, the systematic incorporation of robust errors would enhance the reliability of inference.

 In this regard, the inclusion of the following paragraph is proposed at the end of the **Results Discussion** section:

 Paragraph to be incorporated

 As a methodological limitation, it is acknowledged that estimation using a log-linear OLS model may be subject to heteroskedasticity issues, which are common in income equations. This highlights the importance of systematically employing robust standard errors to strengthen the validity of inference. Additionally, although this study adopts annual cross-sections using the Mincer equation with selection correction, future research could explore panel data model variants—when consistent longitudinal identifiers become available—that would allow for capturing unobserved heterogeneity across regions or cohorts, thereby enriching the causal interpretation of the relationship between UCPR and labor income.

**Comment 3 (Integrated)**

***“The key regressor (mobile phone ownership) is potentially endogenous. While the Heckman procedure partially addresses selection bias, it does not resolve endogeneity due to omitted variables, such as digital skills or entrepreneurial orientation. This could bias the coefficients upwards or downwards. The use of valid external instruments through IV/2SLS could mitigate this risk. Additionally, the study does not explore the mechanisms through which mobile phone use may influence income—such as access to markets, financial services, or job opportunities—limiting the interpretive depth of the findings.***

*The results are clearly presented year by year, highlighting temporal trends. However, coefficient instability is evident, with UCPR estimates ranging from 16.4% in 2017 to 49.9% in 2022. Such variability suggests model instability or the influence of unobserved shocks. The non-significant effects in 2020 and 2021 are attributed to the pandemic; however, no robustness checks or control variables are included to support this claim. Consolidating the analysis using panel models could help disentangle structural effects from year-specific shocks.”*

 In response, the inclusion of the following paragraph is proposed at the end of the **Results Discussion** section, following the limitations of the study:

 Paragraph to be incorporated

 As a projection for future research, it is recommended to address more thoroughly the potential endogeneity of mobile phone ownership through the implementation of instrumental variable strategies or omitted variable control techniques, provided that suitable exogenous instruments are available in comparable datasets. Additionally, it is relevant to explore the mechanisms through which mobile phone use impacts labor income—particularly through access to goods and services markets, digital financial inclusion, or the expansion of employment opportunities. This would enhance the understanding of the observed relationship and strengthen the explanatory power of the results.

**Comment 4**

***“Some effect sizes, particularly in 2022 (0.49), are relatively large and warrant a more detailed analysis of potentially omitted factors. The inclusion of marginal effect interpretations—especially for binary regressors—would enhance the practical relevance of the results by translating statistical findings into income estimates.”***

 In response to this comment, it is clarified that the analysis presented in Table 5 provides a detailed interpretation of the estimated effect, explicitly considering the implications of a binary change from 0 to 1, as well as the interpretation of marginal effects. This approach enhances the practical relevance of the findings by translating the statistical associations into economically meaningful insights.

**Comment on Bibliography and References**

*“ **Although the study cites recent and geographically relevant literature on ICTs and rural development, it underutilizes specialized econometric sources directly related to the Mincer equation and the Heckman model. Foundational works such as Wooldridge (2010), Cameron and Trivedi (2005), and Card (1999) are absent, which limits the methodological foundation. The reference to Mincer (1996) lacks a complete bibliographic entry and appears to rely on an uncommon or imprecisely adapted version instead of the canonical 1974 work.”***

 In response, the inclusion of the following paragraph is proposed at the end of the **Methods** section, prior to the **Methodological Design** subsection:

 Paragraph to be incorporated

 To support the econometric strategy, we rely on the canonical formulation of Mincer (1996), which establishes a log-linear relationship between income, education, and work experience. This approach, widely adopted in the labor economics literature, is implemented through an income equation complemented by the sample selection correction proposed by Heckman (1979), in order to avoid selection bias. In line with advanced econometric guidelines, this two-step procedure addresses issues related to censored or missing data and enhances causal identification. Additionally, following the recommendations of Cameron & Trivedi (2005) regarding robust inference in microeconometrics, we report robust standard errors to correct for the heteroskedasticity typically present in income data. Nevertheless, we acknowledge that mobile phone ownership may be endogenous within the model; consistent with the caution raised by Card (1999) regarding the causal interpretation of returns to education, we consider the potential use of instrumental variables in future extensions to mitigate omitted variable bias. By integrating these foundational works (Cameron & Trivedi, 2005; Wooldridge, 2010) and explicitly citing the seminal work of Mincer (1996) in place of non-standard references, we strengthen the methodological foundation of the study and align our approach with classical econometric theory.

**As a result of the aforementioned adjustments, the following bibliographic references should be incorporated.**

**References**

 Cameron, C., & Trivedi, P. (2005). *Microeconometrics: Methods and Applications - A. Colin Cameron, Pravin K. Trivedi - Google Libros* (1, Ed.; 1st ed.). Cambrigue University Press. https://url-shortener.me/4F0P

 Card, D. (1999). The Causal Effect of Education on Earnings. *Handbook of Labor Economics*, *3*(1), 1801–1863. https://doi.org/10.1016/S1573-4463(99)03011-4

 Wooldridge, J. M. (2010). *Introducción a la econometría. Un enforque moderno* (Vol. 1). www.FreeLibros.me

# Reviewer report: Reviewer response for version 1

**DOI:** 10.5256/f1000research.167511.r397779

**Published:** 2025-08-23

**Authors:** Rosse Marie Esparza Huamanchumo

**Affiliations:** 1 Universidad San Ignacio de Loyola, Lima District, Lima Region, Peru

I congratulate the authors for conducting this research, which aims to analyze the relationship between mobile phone ownership and the income of rural women in Peru, using data from the National Household Survey (ENAHO) for the period 2016-2022. This is a relevant and timely study that addresses a social and economic issue of great impact from a rigorous and well-founded technical approach.

 Although the article is solid and meets the standards of scientific research, some minor adjustments are recommended to enhance its quality prior to publication.

1. **Improve the literature review:** it is suggested to incorporate more recent studies (2023-2025) and organize the state of the art according to the main variables and dimensions of the study. This will allow for a more structured and up-to-date reading of the conceptual framework.
2. **Strengthen the debate on causality:** despite the appropriate use of the Heckman method to correct for selection bias, it would be pertinent to include a more critical reflection on the limitations of the study when establishing direct causal relationships. This would bring greater transparency and rigor to the analysis.
3. **Specify the implications for public policy:** Although the Sustainable Development Goals (SDGs) framework is mentioned, it would be valuable to propose more concrete recommendations aimed at the implementation of public policies on digital inclusion, gender equality, and technological literacy in rural areas.

These adjustments will enhance its academic contribution.

Is the work clearly and accurately presented and does it cite the current literature?

Partly

If applicable, is the statistical analysis and its interpretation appropriate?

Yes

Are all the source data underlying the results available to ensure full reproducibility?

Yes

Is the study design appropriate and is the work technically sound?

Yes

Are the conclusions drawn adequately supported by the results?

Yes

Are sufficient details of methods and analysis provided to allow replication by others?

Yes

Reviewer Expertise:

Entrepreneurship, sustainable territorial development, tourism, and gastronomy

I confirm that I have read this submission and believe that I have an appropriate level of expertise to confirm that it is of an acceptable scientific standard.

# Response

**Published:** 2025-08-30

**Authors:** Lindon Vela Meléndez

**Affiliations:** Lambayeque, Universidad Nacional Pedro Ruiz Gallo, Lambayeque, Lambayeque, Peru

**The following are responses to the recommendations made to the article.**

**Improve the literature review:**

 Before the last paragraph of the introduction, include the following:

 In recent years, various studies have emphasized the persistence of the gender digital divide in rural contexts of low- and middle-income countries, even amid increasing mobile technology adoption. For example, the *Mobile Gender Gap Report 2023* by GSMA reveals that women in low- and middle-income countries are 7% less likely than men to own a mobile phone and 19% less likely to use mobile internet, with rural women and those with lower educational attainment being the most affected (Jeffrie, 2023). In Peru, Ingunza (2023) finds that mobile phone adoption among rural women is positively associated with income levels, using models inspired by Mincer’s earnings equation (Ingunza, 2023). These findings underscore the need to structure the study’s theoretical framework around three core dimensions: (1) the gender digital divide in rural settings; (2) the relationship between mobile phone ownership and women’s economic empowerment; and (3) the structural factors such as education, connectivity, and sociocultural norms that mediate this relationship.

**Strengthen the causality discussion:**

 This should be included as the final paragraph under the limitations section:

 Although the Heckman selection model partially mitigates selection bias, it does not ensure full causal inference, especially in the presence of omitted variables such as digital skills, quality of connectivity, or the productive use of mobile phones. Recent studies have reinforced the need to complement this approach with more robust methodologies. For instance, research in Bangladesh by Matsuura-Kannari et al. (2024) has combined fixed-effects models with panel data to control for unobserved heterogeneity and estimate the impact of mobile phone use on income diversification and poverty reduction. Moreover, reports such as *The Mobile Gender Gap Report 2024* highlight that gender disparities in mobile ownership and usage persist, suggesting that ownership does not automatically translate into economic empowerment, particularly in rural areas. In light of this, future research is encouraged to consider: (a) longitudinal panel data, to capture more accurate temporal and causal dynamics; (b) propensity score matching (PSM), to balance observable covariates; and (c) valid instrumental variables, such as variations in network coverage or targeted technological interventions, to approximate more reliable causal estimates.

**Specify policy implications:**

 This should be added as the final paragraph of the discussion section:

 Within the framework of the Sustainable Development Goals particularly SDG 5 (Gender Equality), SDG 8 (Decent and Inclusive Work), SDG 9 (Industry, Innovation, and Infrastructure), and SDG 10 (Reduced Inequalities) the findings of this study can inform concrete public policy recommendations. These include: (a) subsidized access programs for mobile devices and internet services in rural areas, prioritizing women; (b) targeted digital training for rural women, focused on the productive use of mobile phones in agriculture, commerce, or finance; (c) multisectoral institutional alignment, promoting partnerships among the Ministries of Women, Agricultural Development, Transport, and local governments to integrate mobile solutions into rural development strategies; and (d) incentives for telecommunications companies to develop relevant content and services tailored for rural women. These recommendations not only address the gaps identified in the study but also offer practical pathways toward equitable digital inclusion in Peru.

**New references**

*To be included in alphabetical order in the reference list.*

 Ingunza, F. (2023). Rural women and the use of mobile phones in Peru. Effect on empowerment with a vision of human capital. *Lecturas de Economia*, *98*(98). https://doi.org/10.17533/udea.le.n98a348118

 Jeffrie, N. (2023). The Mobile Gender Gap Report 2022. In *Gsma* (p. 65). https://www.gsma.com/mobilefordevelopment/blog/the-mobile-gender-gap-report-2022/

 Matsuura-Kannari, M., Islam, A. H. M. S., & Tauseef, S. (2024). Mobile phones, income diversification, and poverty reduction in rural Bangladesh. *Review of Development Economics*, *28*(4), 1475–1493. https://doi.org/10.1111/RODE.13110;JOURNAL:JOURNAL:14679361
